# Early right ventricular systolic dysfunction in patients with systemic sclerosis without pulmonary hypertension: a Doppler Tissue and Speckle Tracking echocardiography study

**DOI:** 10.1186/1476-7120-8-3

**Published:** 2010-01-22

**Authors:** Sebastian Schattke, Fabian Knebel, Andrea Grohmann, Henryk Dreger, Friederike Kmezik, Gabriela Riemekasten, Gert Baumann, Adrian C Borges

**Affiliations:** 1 Universitätsmedizin Berlin, Medical Clinic for Cardiology and Angiology, Charité Campus Mitte, Charitéplatz 1, 10117 Berlin, Germany; 2 Universitätsmedizin Berlin, Medical Clinic for Rheumatology, Charité Campus Mitte, Charitéplatz 1, 10117 Berlin, Germany

## Abstract

**Background:**

Isovolumetric acceleration (IVA) is a novel tissue Doppler parameter for the assessment of systolic function. The aim of this study was to evaluate IVA as an early parameter for the detection of right ventricular (RV) systolic dysfunction in patients with systemic sclerosis (SSc) without pulmonary hypertension.

**Methods:**

22 patients and 22 gender- and age-matched healthy subjects underwent standard echocardiography with tissue Doppler imaging (TDI) and speckle tracking strain to assess RV function.

**Results:**

Tricuspid annular plane systolic excursion (TAPSE) (23.2 ± 4.1 mm vs. 26.5 ± 2.9 mm, p < 0.006), peak myocardial systolic velocity (Sm) (11.6 ± 2.3 cm/s vs. 13.9 ± 2.7 cm/s, p = 0.005), isovolumetric contraction velocity (IVV) (10.3 ± 3 cm/s vs. 14.8 ± 3 cm/s, p < 0.001) and IVA (2.3 ± 0.4 m/s^2 ^vs. 4.1 ± 0.8 m/s^2^, p < 0.001) were significant lower in the patient group. IVA was the best parameter to predict early systolic dysfunction with an area under the curve of 0.988.

**Conclusion:**

IVA is a useful tool with high-predictive power to detect early right ventricular systolic impairment in patients with SSc and without pulmonary hypertension.

## Background

Systemic sclerosis is a connective tissue disease characterized by vascular inflammation and fibrosis. Visceral involvement, e.g. pulmonary fibrosis, pulmonary hypertension, myocardial and renal affliction, is associated with poor prognosis [1-4]. Cardiac manifestations include myocardial fibrosis, hypertrophy, coronary and conduction systems disorders that can lead to severe clinical complications such as congestive heart failure, arrhythmias and sudden cardiac death [5].

Primary myocardial involvement and pulmonary hypertension are common in SSc. According to histological and clinical studies, cardiac involvement occurs in up to 80% of SSc patients [3,6-8]. It often begins in early stages of the disease and initially remains clinically asymptomatic. Therefore, early detection of cardiac involvement in patients with SSc is of clinical importance to identify patients with higher risk which would benefit from early medical intervention. Impaired hemodynamic parameters of right ventricular (RV) function are predictors of poor outcome in patients with SSc. A good correlation of RV myocardial diastolic dysfunction and increased pulmonary artery systolic pressure has been demonstrated [9]. However, pulmonary artery pressure did not correlate well with impaired RV systolic function indicating RV systolic dysfunction to be the result of pulmonary hypertension, disturbance of myocardial microcirculation and myocardial fibrosis [10]. These findings suggest that RV diastolic dysfunction is the first detectable myocardial manifestation in SSc.

Novel echocardiographic approaches like Tissue Doppler Imaging and Speckle Tracking Strain Imaging improved the assessment of myocardial systolic and diastolic function in both left and right ventricle and therefore led to better detection of subclinical cardiac involvement in patients with SSc [11,12]. Previous studies have shown that left ventricular hypertrophy and diastolic dysfunction often occur in patients with SSc, while systolic function is frequently only slightly impaired or normal [13-16].

Isovolumetric acceleration (IVA) is a new tissue Doppler parameter for the assessment of systolic function of both left and right ventricle. IVA is calculated as the ratio of Tissue Doppler derived peak myocardial velocity during isovolumetric contraction (IVV) divided by the acceleration time (AT). This parameter has been validated in a variety of experimental and clinical settings. While IVA remains unaffected by changes in pre- and afterload within the physiological range, it can detect small changes in contractile function [17,18]. First clinical studies have demonstrated IVA as a useful sensitive tool to reveal reduced right ventricular systolic function [19-22]. It may also be used for early detection of right ventricular systolic dysfunction [19].

The aim of this study was to assess right ventricular diastolic and systolic function in patients with SSc without pulmonary hypertension and to evaluate IVA as an early parameter for the detection of systolic dysfunction in these patients.

## Methods

### Study population

Twenty-two patients with SSc (17 women, 5 men, mean age 57 ± 13.4 years, range 31 to 77 years) were included into the study. According to serological antibody analysis and Rodnan Skin Score, 11 patients had a limited form and 11 patients suffered from a diffuse form of SSc. Serological antibody analysis revealed the presence of anti-centromere pattern in 8 patients, and anti Scl-70 in 9 patients. All patients presented with normal pulmonary artery pressure as determined by transtricuspid conventional Doppler echocardiography. Three patients with poor RV images were excluded from the study. For comparison, 22 gender- (17 women, 5 men) and age-matched (mean age 57 ± 13.9 years, range 30 to 78 years) subjects with a clinical indication for routine echocardiography and normal findings were used as control group. All participants provided written consent. The local ethics committee approved the protocol.

### Pulmonary function test

In all patients, a pulmonary function test was performed (Jaeger MasterScreen, Body/Diff Box; Jaeger; Wuerzburg, Germany) according to the recommendations of the American Thoracic Society prior to echocardiography [23].

### Standard echocardiography

Standard echocardiography was performed in the left decubitus position using an ultrasound system (Vivid 7, GE Medical Systems, Horton, Norway) with a 3.4-MHz multifrequency transducer. RV images were obtained from the parasternal short axis and at the modified apical four-chamber view to optimize RV visualization. The two RV outflow tract diameters (RVOT1 and RVOT2) were measured in the two-dimensional parasternal short axis as recommended by the European Association of Echocardiography and American Society of Echocardiography [24]. The RV ejection fraction was estimated by Simpson's rule using the apical four-chamber view. The RV-diameter was also measured in the apical four-chamber view above the tricuspid annular plane. Tricuspid annular plane systolic excursion (TAPSE) as a parameter for RV long axis function was assessed with M-Mode cursor positioned at the free wall angle of the tricuspid valve annulus. From subcostal view the diameter of the inferior vena cava and the collapsibility index was measured. Real-time 2D ultrasound data from the RV free wall with a frame rate greater than 40 frames per second (fps) were recorded at the apical view for offline 2D strain analysis [Additional file 1 and 2].

### Conventional Doppler echocardiography

Tricuspid valve regurgitation was detected at the apical four-chamber view by colour Doppler echocardiography. Transtricuspid retrograde velocities were obtained using continuous-wave Doppler. Systolic pulmonary artery pressure was estimated from the peak pressure gradient calculated from three consecutive beats using the modified Bernoulli formula (ΔP = 4V^2^) and the right atrial pressure derived by the diameter of the inferior vena cava and the collapsibility index [24,25].

### Tissue Doppler imaging and pulsed-wave TDI

**Figure 1:**
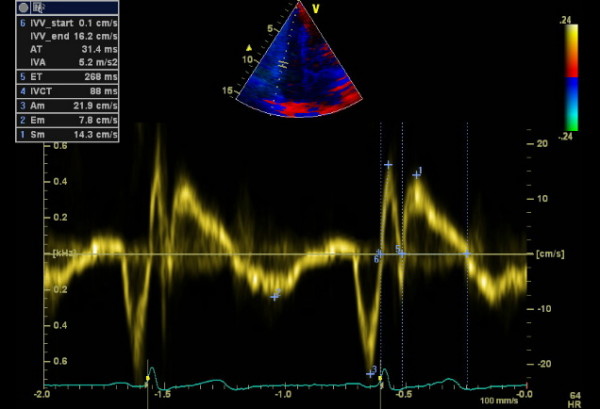
**Pulsed wave tissue Doppler imaging of the RV free wall of a control subject**. 1: peak myocardial systolic velocity (Sm), 2: peak early diastolic velocity (Em), 3: peak late diastolic velocity (Am) 4: isovolumetric contraction time (IVCT), 5: ejection time (ET), 6: peak myocardial isovolumetric contraction velocity (IVV), acceleration time (AT), isovolumetric acceleration (IVA).

**Figure 2:**
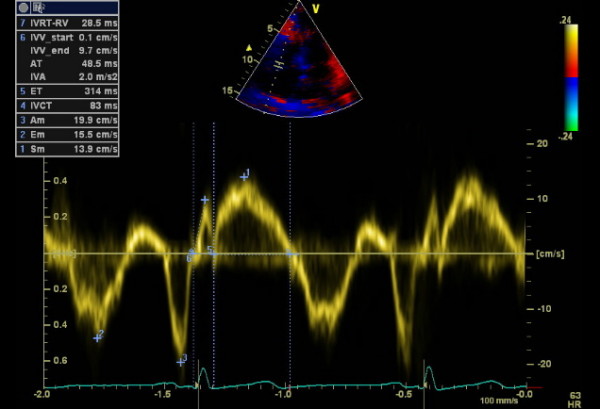
**Pulsed wave tissue Doppler imaging of the RV free wall of an SSc patient**. 1: peak myocardial systolic velocity (Sm), 2: peak early diastolic velocity (Em), 3: peak late diastolic velocity (Am) 4: isovolumetric contraction time (IVCT), 5: ejection time (ET), 6: peak myocardial isovolumetric contraction velocity (IVV), acceleration time (AT), isovolumetric acceleration (IVA), 7: isovolumetric relaxation time (IVRT-RV).

Tissue Doppler Imaging of the RV free wall was performed in the apical four-chamber view at end-expiration. To avoid aliasing, the Nyquist limit was adjusted to the lowest level. Three consecutive cycles were recorded with a frame rate greater than 150 fps for offline strain analysis. Pulsed TDI was performed to measure systolic and diastolic myocardial velocities at the basal level of the RV free wall. Peak myocardial isovolumetric contraction velocity (IVV), peak myocardial systolic velocity (Sm), peak early and late diastolic velocities (Em and Am), isovolumetric contraction time (IVCT), isovolumetric relaxation time (IVRT) and ejection time (ET) were measured [Figures 1 and 2].

### Offline analysis

All images were stored digitally for offline analysis (EchoPac PC Dimension, GE Vingmed). B-Mode images of the RV free wall were used to measure 2D strain using speckle-tracking algorithm. 2D strain in the basal, mid and apical segment of the RV free wall was measured. For TDI analysis, 3 beats were stored. The region of interest (ROI) for TDI strain was placed at basal, mid and apical segment of the RV free wall. TDI strain in each segment was averaged over 3 consecutive cardiac cycles. The myocardial acceleration during isovolumetric contraction (IVA), defined as the ratio of IVV divided by the acceleration time (AT; time to the peak velocity), and the myocardial performance index or Tei index, defined as the ratio between total RV isovolumetric time (IVCT and IVRT) divided by ejection time (ET), were also calculated offline [17,26]. The IVA and AT were measured as follows: The basal and peak points of the IVV curve were marked. The IVA and AT were calculated automatically by EchoPac and/or Vivid7 by a previously programmed algorithm.

### Inter- and intraobserver variability analysis

Three echocardiographers, blinded to clinical data, independently measured IVV and AT and calculated IVA of 10 randomized subjects (five patients and five controls) for interobserver variability analysis. One observer calculated IVA twice for the 10 randomized subjects on two consecutive days for intraobserver variability analysis.

### Statistics

Statistics were calculated by software (SPSS, Version 17.0, SPSS Inc, Chicago, Ill). All results are presented as mean ± standard deviation (SD). The Mann-Whitney nonparametric test was used to compare echocardiographic data from patients and control subjects. Differences were considered statistically significant if the P value was less than 0.05. Receiver-operating characteristic curve analysis was performed to test the diagnostic accuracy for discrimination of patients from controls and to determine optimal cut off values. The correlation was calculated as Pearson's r coefficient to assess univariate relations. Interclass Correlation Coefficient by Kolmogorov-Smirnov was used to calculate inter- and interobserver variability.

## Results

### Clinical characteristics and pulmonary function test

Age, gender, body mass index, systolic and diastolic blood pressure as well as the incidence of cardiovascular risk factors such as hypertension, hypercholesterolemia and diabetes mellitus did not differ significantly between patient and control group [Table 1]. The values for diffusion capacity of the lung for carbon monoxide (DLCO) are presented as a percentage of the predicted value adjusted for age and hemoglobin. The mean DLCO value was significantly reduced in the scleroderma group (63% of predicted value). In 10 patients with diffuse form (91%) and 8 patients with limited form (73%) the DLCO was less than 80% of the predicted value. The mean systolic pulmonary artery pressure derived by transtricuspid pressure gradient was 23 ± 4 mmHg in the SSc group.

**Table 1:** Clinical characteristics, data are expressed as mean ± SD, except gender, hypertension, hypercholesterolemia and diabetes mellitus.

	Scleroderma (n = 22)	Control (n = 22)	p value
Age	57 ± 13.4	57 ± 13.9	0.90

Gender (female)	17	17	1.00

Systolic BP, mmHg	134 ± 21.1	132 ± 16	0.83

Diastolic BP, mmHg	79 ± 8.1	84 ± 9.6	0.17

Body Mass Index, kg/m^2^	24.7 ± 5.1	23.3 ± 2.9	0.44

Hypertension	11	13	0.545

Hypercholesterolemia	7	5	0.498

Diabetes mellitus	3	2	0.635

### LV ejection fraction, RV-dimensions and systolic function at conventional echocardiography

LV ejection fraction was similar in both groups (59.1 ± 4.3% vs. 59.6 ± 1.5%, p = 0.607). There were no significant differences in RVOT1 (29.2 ± 4 mm vs. 28.7 ± 3.4 mm, p = 0.953), RVOT2 (19.7 ± 2.5 mm vs. 20.1 ± 3.3 mm, p = 0.559), RV diameter (31.3 ± 3.5 mm vs. 29.9 ± 2.6 mm, p = 0.184) and RVEF (55.4 ± 12.3% vs. 58.2 ± 8.2%, p = 0.533) between patients and control subjects. TAPSE as a parameter for RV systolic function measured in M-mode was significantly smaller in patients with SSc compared to the control subjects (23.2 ± 4.1 mm vs. 26.5 ± 2.9 mm, p = 0.006).

### RV-two-dimensional and tissue Doppler-derived Strain

In speckle tracking strain analysis, peak systolic strain values were significantly different between both groups in the basal and medial segments of the RV free lateral wall with higher values in the control group (-21.6 ± 8.9% vs. -28.2 ± 5.9%, p = 0.024 and -22.4 ± 8% vs. -29.1 ± 5.8%, p = 0.014 respectively). However, there was no significant difference in the peak systolic strain in the apical segment (-23.2 ± 8.5% vs. -27.6 ± 4.6%, p = 0.106). In six controls and three patients, basal and medial 2D-strain was not measurable due to poor tracking. Apical 2D-strain could not be evaluated in five controls and in two patients respectively. DTI-derived peak systolic strain analysis showed significant differences in all segments of the RV free lateral wall between the scleroderma and the control group. Only in one patient, DTI-derived strain in the medial segment could not be measured due to echo artefacts and reverberation [Table 2].

**Table 2:** Tissue Doppler-derived peak systolic strain, data are expressed as mean ± SD

	Scleroderma (n = 21)	Control (n = 22)	P value
DTI-Strain basal, %	-24.3 ± 7.2	-28.5 ± 6.4	0.040

DTI-Strain medial, %	-26.6 ± 10	-33.4 ± 7.2	0.003

DTI-Strain apical; %	-25.2 ± 7.9	-33.1 ± 8	0.004

### Pulsed wave TDI

The parameters derived from pulsed wave myocardial TDI are listed in Table 3. Except early diastolic peak velocity, all values were statistically significantly lower in patients with SSc compared to the control group. The most significant differences were seen for IVRT, IVV, IVA and MPI with P value < .001 [Table 3].

**Table 3:** Pulsed- DTI-derived parameters measured at the free wall angle of the tricuspid valve annulus, data are expressed as mean ± SD

	Scleroderma (n = 22)	Control (n = 22)	P value
Sm, cm/s	11.6 ± 2.3	13.9 ± 2.7	0.005

Em, cm/s	11.3 ± 3.4	12.9 ± 3.6	0.128

Am, cm/s	14.1 ± 3.4	19 ± 5.4	0.002

IVV, cm/s	10.3 ± 3	14.8 ± 3	< 0.001

AT, ms	44.6 ± 12.7	37.2 ± 9.8	0.026

IVRT, ms	62.4 ± 34.6	11.7 ± 18.2	< 0.001

IVA, m/s^2^	2.3 ± 0.4	4.1 ± 0.8	< 0.001

MPI	0.55 ± 0.22	0.29 ± 0.09	< 0.001

Sm - peak systolic myocardial velocity, Em - early diastolic myocardial velocity, Am - late diastolic myocardial velocity, IVV - isovolumetric myocardial velocity, AT - acceleration time, IVRT - isovolumetric relaxation time, IVA - isovolumetric acceleration, MPI - myocardial performance index

### Comparison between diffuse and limited type of SSc

There are no significant differences among the echo parameters in patients with diffuse and limited type of SSc [Table 4].

**Table 4:** Comparison between scleroderma subtypes, data are expressed as mean ± SD

	Diffuse-type SSc Scleroderma (n = 11)	Limited-type Scleroderma (n = 11)	P value
Systolic PAP, mmHg	23 ± 6.5	23 ± 2.7	0.562

DLCO, %	56.6 ± 16.5	70.1 ± 13.8	0.065

RVOT 1, mm	29.1 ± 4.3	29.3 ± 3.9	0.847

RVOT 2, mm	20.5 ± 2.5	18.7 ± 2.3	0.114

RV-Diameter, mm	31.2 ± 2.9	31.4 ± 4.2	0.949

RVEF, %	49.9 ± 8.5	52.4 ± 8.8	0.562

TAPSE, mm	22.6 ± 4.6	23.8 ± 3.8	0.591

Sm, cm/s	11.6 ± 2.7	11.5 ± 2.1	1.000

Em, cm/s	10.3 ± 3	12.3 ± 3.7	0.243

Am, cm/s	13.4 ± 3.9	14.7 ± 2.9	0.300

IVV, cm/s	9.7 ± 3.3	10.9 ± 2.7	0.401

AT, ms	44.3 ± 14.3	45 ± 11.5	0.898

IVRT, ms	67 ± 37.8	58 ± 32.3	0.606

IVA, m/s^2^	2.2 ± 0.4	2.4 ± 0.4	0.171

MPI	0.61 ± 0.28	0.49 ± 0.12	0.365

2D-Strain basal, %	-19.9 ± 7.7	-23.1 ± 10	0.661

2D-Strain medial, %	-21.2 ± 6.9	-23.6 ± 9.2	0.604

2D-Strain apical, %	-21.8 ± 9.9	-24.4 ± 7.6	0.656

DTI-Strain basal, %	-24.5 ± 8.5	-24.1 ± 6.2	0.847

DTI-Strain medial, %	-22.7 ± 7.9	-30.8 ± 10.7	0.173

DTI-Strain apical; %	-25.2 ± 7.6	-25.1 ± 8.5	0.797

PAP - pulmonary artery pressure, DLCO - diffusing capacity of the lung for carbon monoxide, RVOT - right ventricular outflow tract, RV-diameter - right ventricular diameter, RVEF - right ventricular ejection fraction, TAPSE - tricuspid annular plane systolic excursion, Sm - peak systolic myocardial velocity, Em - early diastolic myocardial velocity, Am - late diastolic myocardial velocity, IVV - isovolumetric myocardial velocity, AT - acceleration time, IVRT - isovolumetric relaxation time, IVA - isovolumetric acceleration, MPI - myocardial performance index

### Receiver operating characteristic analysis

**Figure 3:**
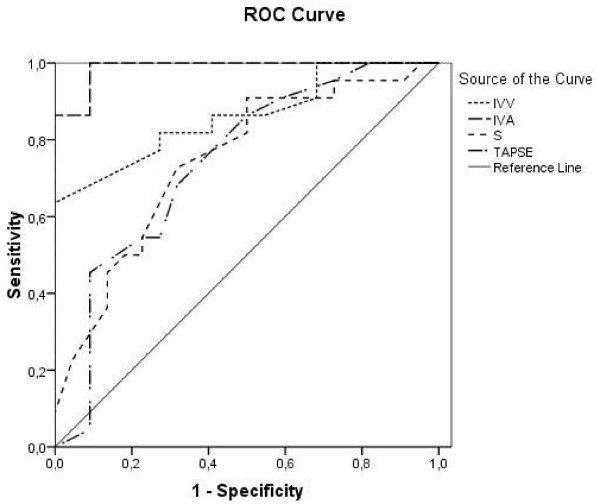
**Receiver operating characteristic curve analysis for IVV - isovolumetric myocardial velocity, IVA - isovolumetric acceleration, S - peak systolic myocardial velocity and TAPSE - tricuspid annular plane systolic excursion**. IVA shows the best area under the curve (0.988) to predict early systolic dysfunction.

Receiver operating characteristic curve analysis was performed to calculate the best cut-off value to predict early systolic and diastolic right ventricular dysfunction in patients with SSc and normal pulmonary arterial pressure. The best parameter to predict early systolic dysfunction was IVA with an area under the curve (AUC) of 0.988. An IVA value less than 3.0 m/s^2 ^had a sensitivity of 1.0 and a specificity of 0.91 to predict early systolic right ventricular dysfunction with a positive predictive value and negative predictive value of 1.0 and 0.92, respectively. The AUC for IVV was 0.861, the peak systolic myocardial velocity (Sm) had an AUC of 0.747 and TAPSE had an AUC of 0.742 [Figure 3]. For speckle tracking derived peak systolic strain in the basal, medial and apical segment the AUC was 0.708, 0.731 and 0.663, respectively. Tissue Doppler derived strain had an AUC in the basal segment of 0.776, in the medial segment of 0.74 and in the apical segment of 0.743. IVRT, a parameter for diastolic dysfunction, had the best AUC with 0.879. An IVRT of 35 ms in the basal segment of the free lateral wall had a sensitivity of 0.77 and a specificity of 0.86 to discriminate patients with SSc and RV diastolic dysfunction with a positive predictive value of 0.81 and negative predictive value of 0.78. The AUC for early diastolic velocities (Em) was 0.633 and for late diastolic velocities (Am) 0.771. The myocardial performance index (MPI) had an AUC of 0.88.

### Correlation of DLCO and RV systolic function

There was no correlation between early-impaired RV systolic function determined by reduced IVA and the degree of impaired DLCO in the pulmonary function test (R^2 ^Linear = 0.013).

### Inter- and intraobserver variability

Inter- and intraobserver variability were found to be good for IVA with an intraclass correlation coefficient of 0.97 (CI 0.94-0.99), p < 0.0001 and 0.98 (CI 0.97-0.99), p < 0.0001.

## Discussion

We could demonstrate that in patients with SSc without pulmonary hypertension systolic and diastolic RV function was impaired. These findings were independent from the subtype of disease.

Only three patients could not be included in this study because of poor RV images. This indicates an acceptable feasibility of echocardiographic assessment of RV function in SSc patients.

Tissue Doppler derived peak systolic velocities and strain as well as two dimensional strain and TAPSE are useful tools to determine early right ventricular systolic dysfunction in patients with SSc without Doppler signs of pulmonary arterial hypertension whereas right ventricular dimension and ejection fraction did not differ significantly from the control group. Even though TAPSE and peak systolic velocity (Sm) were significantly lower compared to the controls in this study, their respective values were still within the normal range according to previously published studies [27-31].

Two-dimensional strain is a new Doppler independent approach for calculation of strain, strain rate, tissue velocity, and displacement. It is based on speckle tracking analysis in B-Mode and therefore angle independent [32]. Previous studies used this method to assess the RV contractility and could show detailed and objective examination of regional contractile function [33]. We could demonstrate a good detection of regional systolic dysfunction with two-dimensional strain in patients with SSc with acceptable AUC in the ROC analysis. But it has to be critically considered that not all patients and controls were fully assessable due to poor tracking of the right ventricular lateral wall. Therefore, this method seems to be less reliable in clinical practice for RV assessment compared to TDI.

IVA is a new sensitive parameter for ventricular contractile function. This parameter has been evaluated in a variety of experimental and clinical settings [18,19,34-36]. Several studies have shown that small changes in contractile function can be detected by measuring IVA while changes in pre- and afterload within the physiological range did not affect this parameter. In contrast to tissue Doppler derived peak systolic velocities, strain and strain rate, IVA is more robust and relatively pre- and afterload-independent [37]. In earlier clinical studies, it could be shown that IVA measured at the level of RV tricuspid annulus detects reduced right ventricular systolic function in relation to the degree of pulmonary regurgitation in patients after repair of tetralogy of Fallot [21,22]. TDI-derived IVA has been used to assess a reduction in functional reserve of both ventricles in patients after atrial repair of dextrotransposition of the great arteries [23]. IVA is also a reliable, noninvasive parameter to assess the positive effect of continuous positive airway pressure (CPAP) therapy in patients with obstructive sleep apnea syndrome on right ventricular systolic function [38]. It may be used for early detection of right ventricular systolic dysfunction in patients with mitral stenosis without signs of systemic venous congestion [19].

Our findings demonstrate that right ventricular IVA, derived by pulsed TDI at lateral tricuspid annulus, has the highest predictive power with the best AUC for detection of early systolic dysfunction. This parameter has a low inter- and intraobserver variability and may be used as an accurate, non-invasive parameter for assessment of RV systolic function in patients with SSc especially to detect early systolic disturbances. An IVA < 3.0 m/s^2 ^predicted SSc patients with 100% sensitivity and 91% specificity.

More than 85% of the patients in our study showed a reduced DLCO. A decreased DLCO is a good predictor for development of pulmonary hypertension in patients with SSc and may exist years before pulmonary hypertension is diagnosed [1]. In a correlation analysis, we could not find a correlation between IVA and the degree of reduction of DLCO in pulmonary function test. Our findings in principle suggest early RV myocardial affection in these patients independently of pulmonary involvement.

RV function could be affected by an impaired left ventricular function. Hence, left ventricular function was analyzed in this patient population and control group as part of routine echocardiography and was found to be within normal and did not differ between the groups.

Lindqvist et al. could demonstrate an impairment of RV diastolic function with an increase in RV wall thickness and RA area in patients with SSc and normal pulmonary artery pressure. Left ventricular function and right ventricular systolic function were not altered even though pulmonary artery acceleration time was reduced in the patient group [39]. As LV function was not affected, it was difficult for the authors to attribute the abnormalities of RV diastolic function to myocardial fibrosis and ischemia due to intermittent coronary vasospasm alone. They assumed that the most probable cause is mild or early intermittent pulmonary arterial hypertension since Doppler echocardiography may underestimate RV pulmonary pressure calculated from transtricuspid pressure gradient [40-42]. In agreement with Lindqvist et al., we obtained pulmonary arterial pressure from a single measurement at rest. Intermittent pulmonary hypertension most likely induced by slight physical exercise cannot be excluded in our patient population.

Our findings of diastolic RV dysfunction were comparable to the results published by Lindqvist et al. In contrast, we also found significantly reduced systolic right ventricular function as determined by reduced IVA. Systolic myocardial velocity (Sm) and TAPSE were also significantly lower compared to our controls but still in normal range according to previously published studies [27-31]. Therefore, Sm and TAPSE alone are not suitable to predict early systolic dysfunction in patients with SSc but still useful tools in routine echocardiography for RV function assessment to indicate patients with clinical relevant RV systolic dysfunction. IVA is calculated as the ratio of IVV divided by AT. We found higher values for IVV in our control group compared to the results of Linqvist et al. [39]. However, our IVV values in the control group conform to normal values for IVV published by the same author earlier [30].

### Study Limitations

The duration of IVA lies in the range of 20-40 msec. Therefore, even with high frame rate approaches like Tissue Doppler Imaging (130-250 fps), were data are acquired every 4-6 msec., low temporal resolution due to technical limitations might represent a relevant source of error. Like all Doppler measurements, IVA is angle dependent. In malpositioned hearts or transducers when the angle of Doppler signal cannot be aligned optimally significant differences may occur. Furthermore, IVA is age-dependent. The highest IVA will be found in the 2^nd ^decade of life, with a progressive decline of IVA in each following decade [43]. The age range in our study population was wide from 31 to 77 years with a mean age of 57.5 ± 12.9 years. To avoid age-dependent bias, our control group was age-matched with a similar range (mean age 57.4 ± 13.4 years, range 30 to 78 years). It was not part of the study protocol to perform right-heart-catheterization to verify our echocardiographic results with invasively measured pulmonary artery pressure.

## Conclusions

This study demonstrated an early RV systolic and diastolic dysfunction in patients with SSc without Doppler indices for pulmonary hypertension. IVA is a new TDI derived index of contractile function with high sensitivity and specificity. IVA is a useful tool with high-predictive power to detect early right ventricular systolic impairment in these patients. With a low intra- and interobserver variability this parameter is easy to measure and not influenced by loading conditions in the physiological range. In order to detect RV- systolic dysfunction as well as diastolic dysfunction in early stages of SSc, further multicenter studies with larger patient numbers are needed to evaluate this parameter for clinical practice.

## Competing interests

The authors declare that they have no competing interests.

## Authors' contributions

SS and FK equally contributed to the study. SS, FK, ACB, AG, IP, HD, participated in contributions to conception, analysis and interpretation of data. BG has supervised and commented the study. ACB was the supervisor of echo examinations, is head of the echo lab, and contributed by revising the manuscript critically. All authors read and approved the final manuscript.

## Supplementary Material

Additional file 1 **Control subject: 2D longitudinal strain of the right ventricle.** Control subject: 2D longitudinal strain of the right ventricle. The values of the basal, mid and apical segment of the free lateral wall were taken for further analysis. Click here for file

Additional file 2 **SSc patient: 2D longitudinal strain of the right ventricle.** SSc patient: 2D longitudinal strain of the right ventricle. The values of the basal, mid and apical segment of the free lateral wall were taken for further analysis. Click here for file
